# 2-{1-[(2-Nitro­benzene­sulfonamido)­meth­yl]cyclo­hexyl}acetic acid

**DOI:** 10.1107/S160053681105224X

**Published:** 2011-12-10

**Authors:** Nosheen Kanwal, Erum Akbar Hussain, Onur Şahin, Orhan Büyükgüngör

**Affiliations:** a Department of Chemistry, Lahore College for Women University, Lahore 54000, Pakistan; b Department of Physics, Ondokuz Mayıs University, TR-55139 Samsun, Turkey

## Abstract

In the title compound, C_15_H_20_N_2_O_6_S, the C—SO_2_—NH—C torsion angle is 64.54 (14)°. In the mol­ecule, there is a bifurcated N—H⋯(O,O) hydrogen bond, forming *S*(7) rings. In the crystal, inversion dimers are formed *via* O—H⋯O hydrogen bonds involving the carboxyl group, so forming *R*_2_^2^(8) rings. These dimers are further linked *via* pairs of C—H⋯O hydrogen bonds, forming a *C*(6) chain propagating along the *c*-axis direction.

## Related literature

For commercial uses of gabapentin {systematic name: 2-[1-(amino­meth­yl)cyclo­hex­yl]acetic acid}, see: Taylor *et al.* (1998 ▶); Cesena & Calcutt (1999 ▶); Field *et al.* (2000 ▶). For the ability of gabapentin to inhibit voltage-dependent Ca^2+^ channel currents, see: Stefani *et al.* (1998 ▶); Walker & De Waard (1998 ▶); Martin *et al.* (2000 ▶); Sutton *et al.* (2002 ▶). For the graph-set analysis of hydrogen-bond patterns, see: Bernstein *et al.* (1995 ▶). For ring puckering parameters, see: Cremer & Pople (1975 ▶).
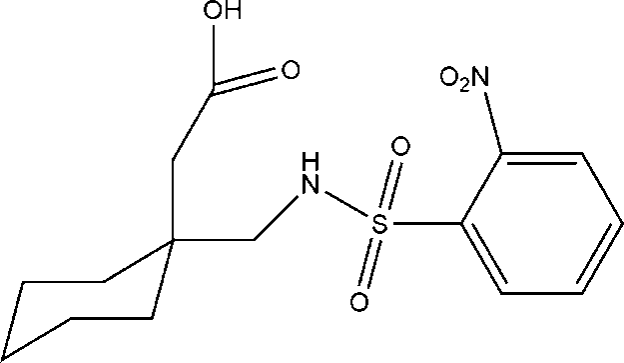


## Experimental

### 

#### Crystal data

C_15_H_20_N_2_O_6_S
*M* *_r_* = 356.39
Monoclinic, 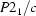

*a* = 7.7383 (2) Å
*b* = 20.7319 (5) Å
*c* = 11.9460 (3) Å
β = 116.869 (1)°
*V* = 1709.59 (7) Å^3^
*Z* = 4
Mo *K*α radiation
μ = 0.22 mm^−1^
*T* = 296 K
0.37 × 0.33 × 0.32 mm

#### Data collection

Bruker APEXII CCD area-detector diffractometer
17069 measured reflections
4247 independent reflections
3202 reflections with *I* > 2σ(*I*)
*R*_int_ = 0.022

#### Refinement

*R*[*F* ^2^ > 2σ(*F* ^2^)] = 0.040
*wR*(*F* ^2^) = 0.110
*S* = 1.02
4247 reflections
221 parameters
H atoms treated by a mixture of independent and constrained refinement
Δρ_max_ = 0.29 e Å^−3^
Δρ_min_ = −0.32 e Å^−3^

### 

Data collection: *APEX2* (Bruker, 2007 ▶); cell refinement: *SAINT* (Bruker, 2007 ▶); data reduction: *SAINT*; program(s) used to solve structure: *SHELXS97* (Sheldrick, 2008 ▶); program(s) used to refine structure: *SHELXL97* (Sheldrick, 2008 ▶); molecular graphics: *ORTEP-3* (Farrugia, 1997 ▶) and *Mercury* (Macrae *et al.*, 2008 ▶); software used to prepare material for publication: *WinGX* (Farrugia, 1999 ▶).

## Supplementary Material

Crystal structure: contains datablock(s) global, I. DOI: 10.1107/S160053681105224X/su2343sup1.cif

Structure factors: contains datablock(s) I. DOI: 10.1107/S160053681105224X/su2343Isup2.hkl

Supplementary material file. DOI: 10.1107/S160053681105224X/su2343Isup3.cml

Additional supplementary materials:  crystallographic information; 3D view; checkCIF report

## Figures and Tables

**Table 1:** Hydrogen-bond geometry (Å, °)

*D*—H⋯*A*	*D*—H	H⋯*A*	*D*⋯*A*	*D*—H⋯*A*
N1—H1⋯O3	0.790 (19)	2.362 (19)	2.978 (2)	135.6 (18)
N1—H1⋯O6	0.790 (19)	2.455 (19)	3.050 (2)	133.0 (18)
O5—H5⋯O6^i^	0.82	1.85	2.6595 (18)	168
C12—H12⋯O2^ii^	0.93	2.50	3.339 (2)	151

Symmetry codes: (i) 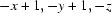; (ii) 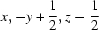.

**Fig. 1.**
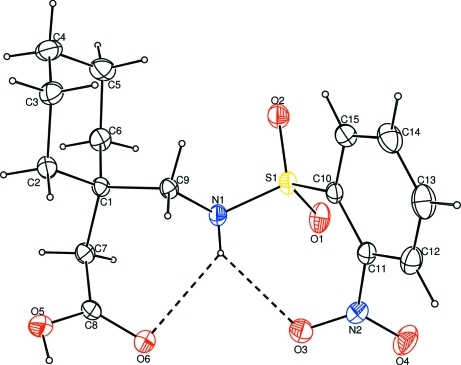
A view of the molecular structure of the title molecule, showing displacement ellipsoids drawn at the 30% probability level. Hydrogen bonds are indicated by dashed lines.

**Fig. 2.**
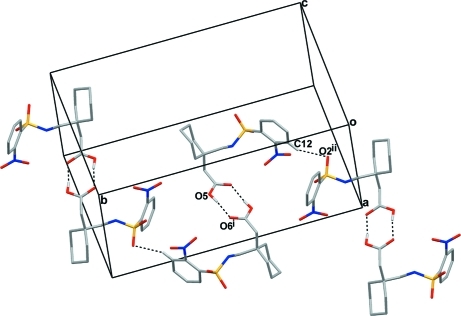
A view of the crystal packing of the title compound, showing the formation of the R22(8) rings, and the C(7) chain. For the sake of clarity, H atoms not involved in these motifs have been omitted [Symmetry codes: (i) -x+1, -y+1, -z; (ii) x, -y+1/2, z-1/2; see Table 1 for further details].

## supplementary crystallographic information

### Comment

The gabapentin, (systematic name: 2-[1-(aminomethyl)cyclohexyl]acetic acid), is used commercially as an anti-convulsant drug and was originally developed for the treatment of spasticity and partial epilepsy (Taylor *et al.*, 1998; Cesena & Calcutt, 1999; Field *et al.*, 2000). Various studies have been undertaken to investigate possible mechanisms of this drug's action. Stefani *et al.* (1998) were the first to demonstrate that gabapentin inhibits voltage-dependent Ca^2+^ channel currents recorded from cortical neurons. This ability of gabapentin to inhibit Ca^2+^ channels has also been demonstrated by number of other groups (Walker & De Waard, 1998; Martin *et al.*, 2000; Sutton *et al.* 2002). However, the drug has poor oral bioavailability and is difficult to synthesize hence, SAR and structural studies on new derivatives of gabapentin is an attractive area of research in medicinal chemistry. Herein, we report on an efficient synthesis and the crystal structure of a new sulfonamide derivative of gabapentin.

The molecular structure of the title compound is shown in Fig. 1. The conformation of the N1—C9 bond in the C—SO_2_—NH—C segment has *gauche* torsions with respect to the S═O bonds. The molecules are twisted at the S1 atom with the C10—S1—N1—C9 torsional angle being 64.54 (14)°. The dihedral angle between the sulfonyl benzene ring and the –SO_2_—NH—C (S1,N1,C9) segment is 86.07 (14)°. The values of the ring puckering parameters: Q_T_ = 0.555 (2) Å, θ = 175.8 (2)° and φ = 328 (3)° (Cremer & Pople, 1975), indicate that the cyclohexane ring has a chair conformation. As shown in Fig. 1 and Table 1, bifurcated intramolecular N1—H1···O3 and N1—H1···O6 hydrogen bonds produce S(7) rings (Bernstein *et al.*, 1995).

In the crystal, hydroxyl O5 acts as a hydrogen-bond donor to the carbonly O atom, O6^i^, so forming an inversion dimer with an *R*_2_^2^(8) ring (Table 1 and Fig. 2). As shown in Fig. 2, these dimers are further linked via a C-H···O interaction, so forming a C(6) chain running parallel to [001].

### Experimental

Gabapentin (0.171 g, 1.00 mmol) was dissolved in distilled water (10 ml) in a round bottom flask (25 ml). The pH of the solution was maintained at 8–9 using 1 *M* Na2CO3 solution. The 2-nitrobenzenesulfonyl chloride (0.221 g, 1.00 mmol) was added to the above solution and stirred at room temperature. The reaction completion was monitored by TLC. Upon completion of the reaction the pH was adjusted 1–2, using 1 *M* HCl solution. The precipitate obtained was filtered, washed with distilled water, dried and recrystallized from methanol to yield colourless crystals.

### Refinement

The imine H atom was located from a difference Fourier map and was refined freely. All other H-atoms were included in calculated positions and refined using a riding model: O-H = 0.82 Å with U_iso_(H) = 1.5U_eq_(O), and C—H = 0.93 and 0.97 Å for H(aromatic) and H(methylene), respectively, with U_iso_(H) = 1.2U_eq_(C).

### Crystal data

**Table:**

C_15_H_20_N_2_O_6_S	*F*(000) = 752
*M**_r_* = 356.39	*D*_x_ = 1.385 Mg m^−^^3^
Monoclinic, *P*2_1_/*c*	Mo *K*α radiation, λ = 0.71073 Å
Hall symbol: -P 2ybc	Cell parameters from 5311 reflections
*a* = 7.7383 (2) Å	θ = 2.9–27.2°
*b* = 20.7319 (5) Å	µ = 0.22 mm^−^^1^
*c* = 11.9460 (3) Å	*T* = 296 K
β = 116.869 (1)°	Block, colourless
*V* = 1709.59 (7) Å^3^	0.37 × 0.33 × 0.32 mm
*Z* = 4	

### Data collection

**Table:**

Bruker APEXII CCD area-detector diffractometer	3202 reflections with *I* > 2σ(*I*)
Radiation source: fine-focus sealed tube	*R*_int_ = 0.022
graphite	θ_max_ = 28.3°, θ_min_ = 2.0°
phi and ω scans	*h* = −10→10
17069 measured reflections	*k* = −27→27
4247 independent reflections	*l* = −15→15

### Refinement

**Table:**

Refinement on *F*^2^	Primary atom site location: structure-invariant direct methods
Least-squares matrix: full	Secondary atom site location: difference Fourier map
*R*[*F*^2^ > 2σ(*F*^2^)] = 0.040	Hydrogen site location: inferred from neighbouring sites
*wR*(*F*^2^) = 0.110	H atoms treated by a mixture of independent and constrained refinement
*S* = 1.02	*w* = 1/[σ^2^(*F*_o_^2^) + (0.0492*P*)^2^ + 0.4452*P*] where *P* = (*F*_o_^2^ + 2*F*_c_^2^)/3
4247 reflections	(Δ/σ)_max_ < 0.001
221 parameters	Δρ_max_ = 0.29 e Å^−^^3^
0 restraints	Δρ_min_ = −0.32 e Å^−^^3^

### Special details

**Table:**

Geometry. All e.s.d.'s (except the e.s.d. in the dihedral angle between two l.s. planes) are estimated using the full covariance matrix. The cell e.s.d.'s are taken into account individually in the estimation of e.s.d.'s in distances, angles and torsion angles; correlations between e.s.d.'s in cell parameters are only used when they are defined by crystal symmetry. An approximate (isotropic) treatment of cell e.s.d.'s is used for estimating e.s.d.'s involving l.s. planes.
Refinement. Refinement of *F*^2^ against ALL reflections. The weighted *R*-factor *wR* and goodness of fit *S* are based on *F*^2^, conventional *R*-factors *R* are based on *F*, with *F* set to zero for negative *F*^2^. The threshold expression of *F*^2^ > σ(*F*^2^) is used only for calculating *R*-factors(gt) *etc*. and is not relevant to the choice of reflections for refinement. *R*-factors based on *F*^2^ are statistically about twice as large as those based on *F*, and *R*- factors based on ALL data will be even larger.

### Fractional atomic coordinates and isotropic or equivalent isotropic displacement parameters (Å^2^)

**Table:**

	*x*	*y*	*z*	*U*_iso_*/*U*_eq_	
C1	0.2548 (2)	0.54510 (7)	0.24581 (13)	0.0348 (3)	
C2	0.1197 (2)	0.59198 (8)	0.14519 (15)	0.0438 (4)	
H2A	0.1787	0.6344	0.1619	0.053*	
H2B	0.1059	0.5781	0.0640	0.053*	
C3	−0.0799 (3)	0.59682 (9)	0.13939 (18)	0.0553 (4)	
H3A	−0.1568	0.6283	0.0765	0.066*	
H3B	−0.1449	0.5554	0.1153	0.066*	
C4	−0.0631 (3)	0.61664 (10)	0.2658 (2)	0.0656 (5)	
H4A	−0.0060	0.6593	0.2871	0.079*	
H4B	−0.1912	0.6185	0.2615	0.079*	
C5	0.0607 (3)	0.56932 (10)	0.36644 (19)	0.0638 (5)	
H5A	−0.0033	0.5277	0.3493	0.077*	
H5B	0.0743	0.5841	0.4471	0.077*	
C6	0.2613 (3)	0.56180 (9)	0.37290 (15)	0.0498 (4)	
H6A	0.3309	0.5281	0.4327	0.060*	
H6B	0.3327	0.6017	0.4036	0.060*	
C7	0.4633 (2)	0.55220 (8)	0.26081 (15)	0.0441 (4)	
H7A	0.5016	0.5971	0.2766	0.053*	
H7B	0.5509	0.5275	0.3333	0.053*	
C8	0.4837 (2)	0.52997 (8)	0.14850 (17)	0.0453 (4)	
C9	0.1812 (2)	0.47606 (7)	0.20560 (14)	0.0371 (3)	
H9A	0.0581	0.4708	0.2075	0.045*	
H9B	0.1595	0.4694	0.1199	0.045*	
C10	0.0958 (2)	0.31795 (7)	0.19092 (14)	0.0377 (3)	
C11	0.1750 (2)	0.27787 (7)	0.13323 (14)	0.0408 (3)	
C12	0.0613 (3)	0.23949 (8)	0.03334 (17)	0.0548 (4)	
H12	0.1174	0.2125	−0.0034	0.066*	
C13	−0.1371 (3)	0.24136 (10)	−0.01188 (19)	0.0648 (5)	
H13	−0.2155	0.2159	−0.0802	0.078*	
C14	−0.2189 (3)	0.28022 (10)	0.0429 (2)	0.0632 (5)	
H14	−0.3528	0.2813	0.0116	0.076*	
C15	−0.1039 (2)	0.31806 (8)	0.14452 (18)	0.0510 (4)	
H15	−0.1610	0.3439	0.1823	0.061*	
N1	0.3172 (2)	0.42711 (6)	0.28645 (14)	0.0441 (3)	
H1	0.398 (3)	0.4174 (9)	0.2660 (18)	0.054 (6)*	
N2	0.3849 (2)	0.27452 (7)	0.17499 (14)	0.0516 (4)	
O1	0.4004 (2)	0.32564 (6)	0.40392 (11)	0.0645 (4)	
O2	0.1074 (2)	0.38599 (6)	0.37366 (13)	0.0643 (4)	
O3	0.47073 (19)	0.32459 (7)	0.18100 (16)	0.0724 (4)	
O4	0.4618 (2)	0.22220 (7)	0.19748 (17)	0.0839 (5)	
O5	0.4434 (2)	0.56977 (6)	0.05920 (13)	0.0641 (4)	
H5	0.4596	0.5525	0.0030	0.096*	
O6	0.5377 (2)	0.47314 (6)	0.14477 (13)	0.0626 (4)	
S1	0.23737 (6)	0.364280 (19)	0.32720 (4)	0.04464 (13)	

### Atomic displacement parameters (Å^2^)

**Table:**

	*U*^11^	*U*^22^	*U*^33^	*U*^12^	*U*^13^	*U*^23^
C1	0.0359 (7)	0.0320 (7)	0.0359 (7)	−0.0062 (6)	0.0158 (6)	−0.0050 (6)
C2	0.0451 (9)	0.0381 (8)	0.0469 (9)	−0.0023 (7)	0.0198 (7)	0.0030 (7)
C3	0.0440 (9)	0.0509 (10)	0.0682 (12)	0.0051 (8)	0.0230 (9)	0.0021 (9)
C4	0.0558 (11)	0.0621 (12)	0.0900 (15)	−0.0012 (9)	0.0428 (11)	−0.0182 (11)
C5	0.0784 (13)	0.0681 (13)	0.0635 (12)	−0.0116 (11)	0.0485 (11)	−0.0197 (10)
C6	0.0591 (10)	0.0487 (9)	0.0400 (8)	−0.0074 (8)	0.0210 (8)	−0.0110 (7)
C7	0.0372 (8)	0.0401 (8)	0.0523 (9)	−0.0092 (6)	0.0177 (7)	−0.0065 (7)
C8	0.0384 (8)	0.0411 (9)	0.0626 (10)	−0.0053 (7)	0.0283 (8)	0.0014 (7)
C9	0.0385 (7)	0.0343 (7)	0.0366 (7)	−0.0066 (6)	0.0152 (6)	−0.0046 (6)
C10	0.0450 (8)	0.0285 (7)	0.0408 (8)	−0.0020 (6)	0.0206 (7)	0.0038 (6)
C11	0.0466 (8)	0.0328 (7)	0.0427 (8)	0.0001 (6)	0.0200 (7)	0.0026 (6)
C12	0.0744 (12)	0.0411 (9)	0.0476 (9)	−0.0029 (8)	0.0265 (9)	−0.0061 (7)
C13	0.0679 (13)	0.0529 (11)	0.0524 (11)	−0.0158 (10)	0.0084 (10)	−0.0034 (9)
C14	0.0453 (10)	0.0577 (12)	0.0723 (13)	−0.0093 (9)	0.0140 (9)	0.0066 (10)
C15	0.0471 (9)	0.0443 (9)	0.0654 (11)	−0.0011 (7)	0.0287 (8)	0.0048 (8)
N1	0.0445 (8)	0.0330 (7)	0.0547 (8)	−0.0023 (6)	0.0223 (7)	0.0002 (6)
N2	0.0531 (8)	0.0444 (8)	0.0598 (9)	0.0074 (7)	0.0277 (7)	−0.0005 (7)
O1	0.0750 (9)	0.0464 (7)	0.0474 (7)	0.0032 (6)	0.0059 (6)	0.0072 (6)
O2	0.1011 (11)	0.0500 (7)	0.0670 (8)	−0.0077 (7)	0.0603 (8)	−0.0054 (6)
O3	0.0548 (8)	0.0564 (8)	0.1159 (13)	−0.0022 (6)	0.0474 (8)	−0.0012 (8)
O4	0.0726 (10)	0.0507 (8)	0.1188 (14)	0.0237 (7)	0.0347 (9)	0.0021 (8)
O5	0.0783 (9)	0.0592 (8)	0.0755 (9)	0.0146 (7)	0.0530 (8)	0.0136 (7)
O6	0.0811 (9)	0.0444 (7)	0.0816 (9)	0.0067 (6)	0.0537 (8)	0.0045 (6)
S1	0.0601 (3)	0.0345 (2)	0.0397 (2)	−0.00341 (17)	0.02283 (19)	0.00003 (15)

### Geometric parameters (Å, °)

**Table:**

C1—C2	1.532 (2)	C9—N1	1.467 (2)
C1—C9	1.5350 (19)	C9—H9A	0.9700
C1—C6	1.536 (2)	C9—H9B	0.9700
C1—C7	1.548 (2)	C10—C15	1.386 (2)
C2—C3	1.518 (2)	C10—C11	1.387 (2)
C2—H2A	0.9700	C10—S1	1.7779 (15)
C2—H2B	0.9700	C11—C12	1.372 (2)
C3—C4	1.513 (3)	C11—N2	1.470 (2)
C3—H3A	0.9700	C12—C13	1.379 (3)
C3—H3B	0.9700	C12—H12	0.9300
C4—C5	1.512 (3)	C13—C14	1.360 (3)
C4—H4A	0.9700	C13—H13	0.9300
C4—H4B	0.9700	C14—C15	1.380 (3)
C5—C6	1.527 (3)	C14—H14	0.9300
C5—H5A	0.9700	C15—H15	0.9300
C5—H5B	0.9700	N1—S1	1.6084 (14)
C6—H6A	0.9700	N1—H1	0.790 (19)
C6—H6B	0.9700	N2—O4	1.2077 (19)
C7—C8	1.493 (2)	N2—O3	1.2167 (19)
C7—H7A	0.9700	O1—S1	1.4244 (13)
C7—H7B	0.9700	O2—S1	1.4240 (13)
C8—O6	1.258 (2)	O5—H5	0.8200
C8—O5	1.271 (2)		
			
C2—C1—C9	108.72 (12)	H7A—C7—H7B	107.7
C2—C1—C6	109.79 (13)	O6—C8—O5	122.54 (16)
C9—C1—C6	111.18 (12)	O6—C8—C7	119.54 (15)
C2—C1—C7	109.67 (12)	O5—C8—C7	117.92 (15)
C9—C1—C7	110.16 (12)	N1—C9—C1	112.64 (12)
C6—C1—C7	107.29 (12)	N1—C9—H9A	109.1
C3—C2—C1	113.41 (13)	C1—C9—H9A	109.1
C3—C2—H2A	108.9	N1—C9—H9B	109.1
C1—C2—H2A	108.9	C1—C9—H9B	109.1
C3—C2—H2B	108.9	H9A—C9—H9B	107.8
C1—C2—H2B	108.9	C15—C10—C11	117.76 (15)
H2A—C2—H2B	107.7	C15—C10—S1	118.65 (13)
C4—C3—C2	110.23 (15)	C11—C10—S1	123.43 (12)
C4—C3—H3A	109.6	C12—C11—C10	121.73 (16)
C2—C3—H3A	109.6	C12—C11—N2	116.35 (15)
C4—C3—H3B	109.6	C10—C11—N2	121.92 (14)
C2—C3—H3B	109.6	C11—C12—C13	119.16 (18)
H3A—C3—H3B	108.1	C11—C12—H12	120.4
C5—C4—C3	110.83 (15)	C13—C12—H12	120.4
C5—C4—H4A	109.5	C14—C13—C12	120.38 (18)
C3—C4—H4A	109.5	C14—C13—H13	119.8
C5—C4—H4B	109.5	C12—C13—H13	119.8
C3—C4—H4B	109.5	C13—C14—C15	120.32 (18)
H4A—C4—H4B	108.1	C13—C14—H14	119.8
C4—C5—C6	111.75 (16)	C15—C14—H14	119.8
C4—C5—H5A	109.3	C14—C15—C10	120.63 (17)
C6—C5—H5A	109.3	C14—C15—H15	119.7
C4—C5—H5B	109.3	C10—C15—H15	119.7
C6—C5—H5B	109.3	C9—N1—S1	120.04 (11)
H5A—C5—H5B	107.9	C9—N1—H1	113.8 (14)
C5—C6—C1	113.24 (14)	S1—N1—H1	110.6 (14)
C5—C6—H6A	108.9	O4—N2—O3	123.53 (16)
C1—C6—H6A	108.9	O4—N2—C11	118.41 (15)
C5—C6—H6B	108.9	O3—N2—C11	118.01 (14)
C1—C6—H6B	108.9	C8—O5—H5	109.5
H6A—C6—H6B	107.7	O2—S1—O1	120.24 (9)
C8—C7—C1	113.23 (12)	O2—S1—N1	107.30 (8)
C8—C7—H7A	108.9	O1—S1—N1	107.49 (8)
C1—C7—H7A	108.9	O2—S1—C10	106.03 (8)
C8—C7—H7B	108.9	O1—S1—C10	106.58 (7)
C1—C7—H7B	108.9	N1—S1—C10	108.81 (7)
			
C9—C1—C2—C3	69.39 (17)	C10—C11—C12—C13	0.8 (3)
C6—C1—C2—C3	−52.45 (18)	N2—C11—C12—C13	−178.76 (16)
C7—C1—C2—C3	−170.10 (13)	C11—C12—C13—C14	−0.8 (3)
C1—C2—C3—C4	57.0 (2)	C12—C13—C14—C15	−0.2 (3)
C2—C3—C4—C5	−57.7 (2)	C13—C14—C15—C10	1.2 (3)
C3—C4—C5—C6	56.3 (2)	C11—C10—C15—C14	−1.1 (2)
C4—C5—C6—C1	−53.1 (2)	S1—C10—C15—C14	−176.65 (14)
C2—C1—C6—C5	50.00 (18)	C1—C9—N1—S1	139.40 (12)
C9—C1—C6—C5	−70.36 (18)	C12—C11—N2—O4	−52.4 (2)
C7—C1—C6—C5	169.12 (15)	C10—C11—N2—O4	128.05 (18)
C2—C1—C7—C8	−67.57 (17)	C12—C11—N2—O3	125.20 (18)
C9—C1—C7—C8	52.07 (17)	C10—C11—N2—O3	−54.3 (2)
C6—C1—C7—C8	173.23 (14)	C9—N1—S1—O2	−49.77 (14)
C1—C7—C8—O6	−94.18 (18)	C9—N1—S1—O1	179.59 (12)
C1—C7—C8—O5	85.21 (18)	C9—N1—S1—C10	64.54 (14)
C2—C1—C9—N1	171.39 (13)	C15—C10—S1—O2	8.75 (15)
C6—C1—C9—N1	−67.63 (17)	C11—C10—S1—O2	−166.48 (13)
C7—C1—C9—N1	51.19 (17)	C15—C10—S1—O1	137.98 (13)
C15—C10—C11—C12	0.2 (2)	C11—C10—S1—O1	−37.26 (15)
S1—C10—C11—C12	175.44 (13)	C15—C10—S1—N1	−106.38 (13)
C15—C10—C11—N2	179.68 (14)	C11—C10—S1—N1	78.38 (14)
S1—C10—C11—N2	−5.0 (2)		

### Hydrogen-bond geometry (Å, °)

**Table:**

*D*—H···*A*	*D*—H	H···*A*	*D*···*A*	*D*—H···*A*
N1—H1···O3	0.790 (19)	2.362 (19)	2.978 (2)	135.6 (18)
N1—H1···O6	0.790 (19)	2.455 (19)	3.050 (2)	133.0 (18)
O5—H5···O6^i^	0.82	1.85	2.6595 (18)	168
C12—H12···O2^ii^	0.93	2.50	3.339 (2)	151

Symmetry codes: (i) −*x*+1, −*y*+1, −*z*; (ii) *x*, −*y*+1/2, *z*−1/2.
